# Generation of diffuse large B cell lymphoma-associated antigen-specific Vα6/Vβ13+T cells by TCR gene transfer

**DOI:** 10.1186/1756-8722-4-2

**Published:** 2011-01-11

**Authors:** Qingsong Yin, Xianfeng Zha, Lijian Yang, Shaohua Chen, Yubing Zhou, Xiuli Wu, Yangqiu Li

**Affiliations:** 1 Institute of Hematology, Medical College, Jinan University; Guangzhou, 510632, PR China; 2 Department of Biochemistry of Medical College, Jinan University, Guangzhou, 510632, PR China; 3 Key Laboratory for Regenerative Medicine of Ministry of Education, Jinan University, Guangzhou, 510632, PR China

## Abstract

**Background:**

Our previous study had amplified antigen-specific full-length TCR α and β genes of clonally expanded T cells in the peripheral blood (PB) of patients with diffuse large B-cell lymphoma (DLBCL). The transfer of T cell receptor (TCR) genes endows T cells with new antigen specificity. Therefore, the aim of this study is to generate diffuse large B cell lymphoma (DLBCL)-specific T cells by T cell receptor (TCR) gene transfer.

**Materials and methods:**

Two different eukaryotic expression plasmids harboring TCR Vα6 and TCR Vβ13 genes specific for DLBCL-associated antigens were constructed and subsequently transferred into human T cells using Nucleofector™ technique. The expression of targeted genes in TCR gene-modified cells was detected by real-time PCR, and western blot using TCR Vβ antibody. The specific cytotoxicity of TCR gene-transferred T cells *in vitro *was estimated using a lactate dehydrogenase (LDH) release assay.

**Results:**

Two different eukaryotic expression plasmids harboring TCR Vα6 and TCR Vβ13 genes specific for DLBCL-associated antigens were constructed and subsequently transferred into T cells from healthy donors. Specific anti-DLBCL cytotoxic T lymphocytes (CTL) could be induced by transduction of specific TCR gene to modify healthy T cells. The transgene cassette of TCR Vβ13-IRES-TCR Vα6 was superior to the other in the function of TCR-redirected T cells.

**Conclusions:**

Specific anti-DLBCL cytotoxic T lymphocyte (CTL) could be inducted by transduction of specific TCR gene to modify healthy T cells.

## Background

In the past two decades, fundamental advances in immunology have introduced cellular-based therapies for cancer patients [1,2]. Donor lymphocyte infusion (DLI) has rendered or induced remission in relapsed patients [3-5]. Autologous tumor-infiltrating lymphocytes (TILs) have been found to mediate objective cancer regression [6-8]. In recent years, specific adoptive immunotherapy with tumor-specific cytotoxic T lymphocyte (CTL) has been considered a promising treatment in malignancy, which might eradicate minimal residual disease without increasing toxicity [9,10]. however, the generation of tumor-specific T cells in this mode of immunotherapy is often limiting. The isolation and *in vitro *expansion of antigen-specific T cell clones remains time-consuming and labor-intensive, such that this treatment is only available to a limited number of patients. To overcome this limitation, another approach has been developed for cancer immunotherapy based on the genetic modification of normal T lymphocytes [11].

Because the molecular basis of CTL specificity is dictated solely by its TCR, which consists of a heterodimeric pair of α- and β-chains (TCRαβ), the molecular transfer of TCR genes from donor to recipient T cells using transgenic technology will result in a transfer of CTL specificity [11,12]. Thus, TCR gene transfer is an attractive strategy for the rapid *in vitro *generation of a high number of antigen-specific T cells [13]. The first TCR gene transfer into primary human T lymphocytes was accomplished with work on melanoma antigen [14] and CD8+T cells transduced with a TCR specific for MART-1 were able to lyse an HLA-A2+melanoma cell line *in vitro*. Subsequently, several other tumor-associated antigens (TAAs) have been selected as targets, such as WT1 protein [15] and P53 protein [16]. In addition, TCR genes specific for HIV and EBV antigens have also been transferred successfully into CD8+T cells from patients [17,18]. In the first clinical trial of TCR gene therapy [19] T cells that had been transduced with a TCR specific for MART-1 mediated some degree of cytotoxicity in 15 patients, demonstrating the feasibility and potential of the anti-tumor effect of TCR gene-modified T cells.

Diffuse large B cell lymphoma (DLBCL) is one of the most common and highly aggressive lymphoid malignancies whose clinical outcomes vary widely. Recently, novel therapeutic strategies, including the incorporation of immunotherapy and combined chemotherapy, have improved the outcome for patients with DLBCL; *e.g*., the combination of rituximab (anti-CD20 antibody) and CHOP regimen (R-CHOP) has been demonstrated to be more effective [20]. Nonetheless, the increased toxicity suggested that novel regimens should be developed to improve long-term disease-free survival. The potential for T cells to contribute to the eradication of B cell malignancies in humans has been illustrated by the ability of allogeneic hematopoietic stem cell transplantation to cure advanced lymphoma, which can be attributed in part to a T cell mediated graft-versus-tumor (GVT) effect. Therefore, much research has focused on the generation of effective antigen-specific T cells. At present, the successful transfer of TCR genes specific for a variety of virus-specific and tumor-associated antigens, such as MART-1/WT1 TCR-modified T cells, has been shown to have specific cytotoxicity on melanoma or leukemia cells [19,21]. However, little is known about the TCR gene-modified T cells specific for lymphoma-associated antigen.

Previously, we found specific TCR gene sequences associated with DLBCL-associated antigen [22] and submitted them to GenBank (Accession numbers: EU369627, EU368854, and so on). In the current study, we developed two types of recombinant constructs containing the HLA-A2-restricted TCR α6 and TCR β13 genes specific for DLBCL-associated antigens with TCR α at either the IRES 5' position or 3' position, which may induce DLBCL-specific T cells by TCR gene transduction. TCR gene-transferred T cells exhibited specific cytotoxicity in response to the DLBCL cell line. Using this approach, we concluded that it is feasible to prepare human tumor-specific T cells from polyclonally activated T cells if we could obtain MHC class I-restricted TCR genes. This strategy will likely lead to individualized immunotherapy based on lymphoma expressing certain proteins in DLBCL.

## Materials and methods

### Construction of recombinant plasmids

**Figure 1:**
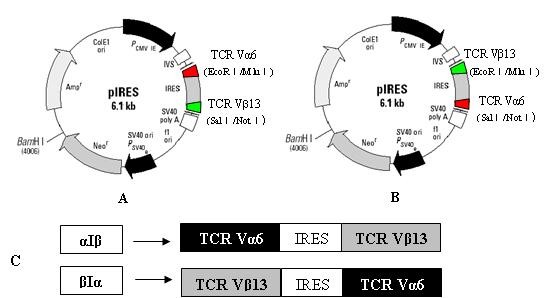
**Schematic representation of plasmid constructs used in the present study**. The structural pattern of two types of expression cassettes of TCR α6- and β13-chain genes. The TCRα- and β-chain genes were introduced into the pIRES vector and linked by an IRES element. For both linker elements, TCRα was integrated into either the 5' position (αIβ) or the 3' position (βIα). A) TCR Vα6-IRES-TCR Vβ13 recombinant plasmid. B) TCR Vβ13-IRES-TCR Vα6 recombinant plasmid. C) The structure of cassettes αIβ and βIα.

DLBCL associated-TCR Vα6 and TCR Vβ13 chain genes, which had been identified in peripheral blood T cells from one DLBCL case were used [22]. Different approaches were applied to construct two recombinant plasmids containing the TCR Vα6- and TCR Vβ13-chain genes specific for DLBCL-associated antigen. Briefly, the full-length TCR α6 and β13 genes specific for DLBCL-associated antigens were amplified by PCR using forward primers (VA6-F, VB13-F) and reverse primers (VA6-R, VB13-R), respectively (Table 1), and subsequently cloned into the eukaryotic expression vector pIRES, respectively, in which the TCR α6- and β13-chain genes were linked by an internal ribosomal entry site (IRES) to construct two different bicistronic eukaryotic expression plasmids (α6-IRES-β13, β13-IRES-α6) with the α- or the β-chain gene in the 5' position and the other gene in the 3' position. The full-length TCRα- and β-chain genes were ligated via EcoR I and Mlu I restriction sites in the 5' position of IRES, and via Sal I and Not I restriction sites in the 3' position. Two kinds of TCR cassettes (Figure 1) were verified by restriction analysis and sequencing.

**Table 1:** The sequence of primers for PCR

Primers	Function	Sequences
Vα6	Sense primer for TCR α6 genes	5'-TCCGCCAACCTTGTCATCTCCGCT-3'
Cα	Antisense primer for TCR α6 genes	5'-GTTGCTCCAGGCCGCGGCACTGTT-3'
Vβ13	Sense primer for TCR β13 genes	5'-CACTGCGGTGTACCCAGGATATGA-3'
Cβ	Antisense primer for TCR β13 genes	5'-CGGGCTGCTCCTTGAGGGGCTGCG-3'
VA6-F	Sense primer for full-length TCR α6 genes	5'-GCCAGGTTCACCTCACAGTACAGAGTCC-3'
VA6-R	Antisense primer for full-length TCR α6 genes	5'-GCAGAGGAAGGAGCGAGGGAGCAC-3'
VB13-F	Sense primer for full-length TCR β13 genes	5'-GCACAGATACAGAAGACCCCTCCGTC-3'
VB13-R	Antisense primer for full-length TCR β13 genes	5'-GGGTGAGGATGAAGAATGACCTGGGATG-3'
VA6-EF	Sense primer for TCR α6 genes in the 5' position of IRES	5'-ACG**GAATTC**GCCAGGTTCACCTCACAGTACAGAG-3'
VA6-MR	Antisense primer for TCR α6 genes in the 5' position of IRES	5'-TCG**ACGCGT***TCA*GAGGAAGGAGCGAGGGAGCAC-3'
VB13-SF	Sense primer for TCR β13 genes in the 3' position of IRES	5'-TTA**GTCGAC**GCACAGATACAGAAGACCCCTCCGTC-3
VB13-NR	Antisense primer for TCR β13 genes in the 3' position of IRES	5-'TAAT**GCGGCCGC***TCA*TGAGGATGAAGAATGACCTGGGATG-3'
VB13-EF	Sense primer for TCR β13 genes in the 5' position of IRES	5'-CG**GAATTC**GCACAGATACAGAAGACCCCTCCGTC-3'
VB13-MR	Antisense primer for TCR β13 genes in the 5' position of IRES	5'-TCC**ACGCGT***TCA*GTGAGGATGAAGAATGACCTGGGATG-3'
VA6-SF	Sense primer for TCR α6 genes in the 3' position of IRES	5'-TTG**GTCGAC**GCCAGGTTCACCTCACAGTACAGAG-3'
VA6-NR	Antisense primer for TCR α6 genes in the 3' position of IRES	5'-TAAT**GCGGCCGC***TCA*GAGGAAGGAGCGAGGGAGCAC-3'

Note: Boldface with underline indicates restriction site; Italic with shadow indicates stop codon.

### Human CD3+T cell isolation and culture

Peripheral blood mononuclear cells (PBMCs) obtained from three healthy donors (HLA-A2, DP restricted) were isolated from heparinized venous blood by Ficoll-Paque gradient centrifugation. All procedures were conducted according to the guidelines of the Medical Ethics Committee of the Health Bureau of Guangdong Province of China. Cells were collected and washed twice in Hank's balanced salt solution, and then finally resuspended at a final concentration of 2 × 10^6 ^cells/mL in complete RPMI 1640 medium (Invitrogen, Grand Island, NY) supplemented with 10% heat-inactivated fetal calf serum (FCS; HyClone, Logan, UT), 100 U/mL penicillin, 100 μg/mL streptomycin, 2 mM L-glutamine, and 50 μM 2-mercaptoethanol. CD3+T cells were positively purified from freshly isolated PBMCs using CD3+microbeads (Miltenyi Biotec, Bergisch Gladbach, Germany) according to the manufacturer's protocol. The purity of collected CD3+T cells was assessed by flow cytometry. More than 95% of CD3+T cells were collected by this technique. Initial stimulation was performed at a concentration of 2 × 10^6 ^cells per well in 1 mL T cell complete medium (+200 IU/mL IL-2 and 2 μg/mL PHA [Sigma, USA]) for 24 h in non-tissue culture 12-well plates. Cells were washed once with medium on the following day and then added to fresh complete RPMI 1640 medium supplemented with 200 IU/ml IL-2. The culture medium was replaced every 2-3 days and the cells were cultured for 5-6 days before transfection to achieve a high transfection efficiency.

### Cell lines and culture

Toledo cells (human diffuse large B cell lymphomas cell line, expressed DLBCL-associated antigen), Molt-4 cells (human acute lymphoblastic leukemia cell line), Raji cells (human Burkitt lymphoma cell line), all from ATCC, were cultured in complete RPMI 1640 medium supplemented with 10% heat-inactivated FBS and maintained at 37°C in a 5% CO_2 _incubator. The medium was replaced every 2-3 days.

### Transduction of TCR genes in T cells

Human CD3+T cells at 6 days after stimulation were transfected using the Nucleofector™ technology (Amaxa, Cologne, Germany). In brief, cells (5 × 10^6^) were resuspended into 0.1 mL supplemented Nucleofector solution at room temperature from the human T cell Nucleofector™ kit. Each plasmid (2 μg; including TCR Vα6-IRES-TCR Vβ13 recombinant plasmid, TCR Vβ13-IRES-TCR Vα6 recombinant plasmid, TCR unloaded plasmid as a negative control, and maxGFP as a positive control) was mixed with 0.1 mL cell suspension and then transferred to a 2.0 mm electroporation cuvette and nucleofected using an Amaxa Nucleofector II apparatus according to the manufacturer's guidelines. Storage of the cell suspension in human T cell Nucleofector solution for longer than 20 min was avoided, as this reduces cell viability and gene transfer efficiency. The cells were transfected using the program T-020. The transfected T cells were transferred immediately to pre-warmed complete culture medium and cultured in 12-well plates in a humidified incubator at 37°C and 5% CO_2_. The culture medium was changed 8 h after transfection to medium containing 200 IU/mL IL-2.

### Determination of transfection efficiency

The transfection efficiency was estimated in each experiment by scoring the number of GFP-positive cells (maxGFP expression) 24 h after transfection. Immunophenotyping analysis was performed using a TCR Vβ13 monoclonal antibody (mAb) with laser confocal microscopy (LCM; 510 META DuoScan, Carl Zeiss, Germany) and by flow cytometry (FCM) 48 h after transfection. Cells were stained with fluorescein isothiocyanate (FITC)-conjugated mAbs. Antibodies were purchased from Beckman Coulter, California, USA (mouse-anti-human TCR Vβ13).

### RNA extraction and cDNA synthesis

Total RNA was extracted from the TCR CD3+T cells that were either gene-transduced or not gene-transduced, according to the manufacturer's recommendations (TRIzol^® ^reagent; Invitrogen, USA). The quality of RNA was analyzed by 0.8% agarose gel electrophoresis with ethidium bromide staining. The RNA (2 μg) was reverse-transcribed into first single-strand cDNA using random hexamer primers, reverse transcriptase, and the Superscript II kit (PowerScript™ Reverse, BD, USA) according to the manufacturer's instructions. The quality of cDNA was confirmed by reverse transcriptase polymerase chain reaction (RT-PCR) for β2 microglubin gene amplification.

### Real-time PCR

The mRNA expressions of antigen-specific TCR Vα6 and TCR Vβ13 genes were detected by real-time PCR using SYBR^® ^Green I with the Real Master Mix kit (Tiangen, Beijing, China). Reactions were run in triplicate and repeated in three independent experiments using the MJ Research real-time PCR system (Bio-Rad, USA) with a cDNA template in a 25 μL reaction under the following conditions: 95°C for 2 min, followed by 45 cycles of 95°C for 15 s and 62°C for 1 min. The primers used in the real-time PCR are listed in Table 1. Similar manipulation was performed with RNA as the template to exclude the presence of plasmid DNA.

### Western blot analysis

CD3+T cells at 2 × 10^6 ^were harvested 3 days after transfection, mixed with RIPA lysis buffer (1 × PBS, 1% Nonidet P-40, 0.5% sodium deoxycholate, 0.1% sodium dodecyl sulfate [SDS], 10 mmol/L phenylmethylsulfonyl fluoride, 1 μg/mL aprotinin, and 100 mmol/L sodium orthovanadate), and incubated on ice for 30 min to isolate total proteins. Proteins (100 μg) were separated by 7.5% SDS-PAGE and transferred to nitrocellulose membranes (Invitrogen, USA) using a damp-dry transfer device (Bio-rad, USA). After blocking for 1 h in 5% defatted milk powder in PBS, the membrane was washed and then probed with 1:300 mouse-anti-human TCR Vβ13 monoclonal antibody (Beckman Coulter, USA). Similar studies were performed with 1:500 mouse-anti-human β actin (BOSTER, Wuhan, China). The antibodies were detected using 1:10000 horseradish peroxidase-conjugated rabbit-anti-mouse IgG (Tiangen, Beijing, China). A Western blotting luminol reagent (Tiangen, Beijing, China) was used to visualize the bands corresponding to each antibody.

### Cytotoxicity assay

TCR gene-transferred CD3+T cells (effector cells) or TCR CD3+T cells that were not gene-transduced were incubated with Toledo cells (DLBCL cell line, ATCC, target cells), Molt-4 cells, or Raji cells in U-bottomed, 96-well microplates at 10:1 effector-target ratios for 10 h at 37°C and 5% CO_2 _in culture media containing 5% FBS for the *in vitro *cytotoxicity assay. Each effector-target mixed condition was analyzed in triplicate and repeated in three independent experiments (transferred CD3+T cells from three healthy individuals). Cell-mediated cytotoxicity was determined using a nonradioactive lactate dehydrogenase (LDH) release assay (Roche, Germany) according to the manufacture's instructions. Spontaneous LDH release from both target and effector cells was subtracted from the measured values and the final results were expressed as a percentage of specific cytotoxicity. Percentage specific lysis was calculated from LDH as follows: (experimental release-target spontaneous release-effector spontaneous release)/(target maximum release-target spontaneous release). The Mann-Whitney test was used to determine differences between two independent samples in cytotoxicity assays. Statistical significance was defined as *P *< 0.05.

## Results

### TCR plasmid construction

In our previous study, expanded TCR Vα and TCR Vβ subfamily T cells were identified in the PB of patients with DLBCL, which was presumed to have been driven by the stimulation of epitopes in DLBCL [22]. The full-length TCRVα6- and Vβ13-chain genes specific for DLBCL-associated antigen had been amplified for the construction of bicistronic recombinant plasmids. Because a gene inserted downstream of the IRES will be expressed at a significantly lower level than one introduced upstream [23] and the two chains may play nonequivalent roles in antigen selection, we integrated the TCRα gene into either the IRES 5' position (TCR Vα6-IRES-TCR Vβ13) or the 3' position (TCR Vβ13-IRES-TCR Vα6), generating two types of recombinant plasmids. Subsequently, their sequence and reading frame were confirmed by restriction enzyme digestion analysis and sequencing (data not shown).

### TCR gene-modified CD3+T cells

**Figure 2:**
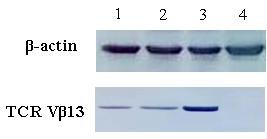
**TCR Vβ13 protein expression was detected in TCR gene-transfected CD3+T cells by Western Blot analysis**. *Lane 1*: CD3+T cells transfected with TCR Vα6-IRES-TCR Vβ13 recombinant plasmid; *Lane 2*: CD3+T cells transfected with TCR Vβ13-IRES-TCR Vα6 recombinant plasmid; *Lane 3*: mononuclear cells from cord blood expressing TCR Vβ13 protein as a positive control; *Lane 4*: CD3+T cells transfected with empty plasmid.

The CD3+T cells were transfected with the respective TCR-encoding expression plasmids using Nucleofector™ technology. After gene transfection, higher V expression levels of Vα6 and Vβ13 gene were detected by real-time PCR respectively(data not shown), and the corresponding protein of TCR Vβ13 chain was detected by SDS-PAGE (Figure 2), because it is difficult to purchase the human TCR Vα subfamily antibodies in China, in this study, we used the Vβ13 antibody to confirm the expression of transfected recombinant plasmid, by combining the results from real-time PCR, it could be concluded that antigen-specific TCR α6 and β13 genes were well expressed in both types of transgene cassettes, indicating that the recombinant plasmids had been constructed successfully and that the expression of exogenous TCR α and β genes could be detected in the transduced cells.

### Influence of the transgene cassette on TCR expression level in CD3+T cells

**Figure 3:**
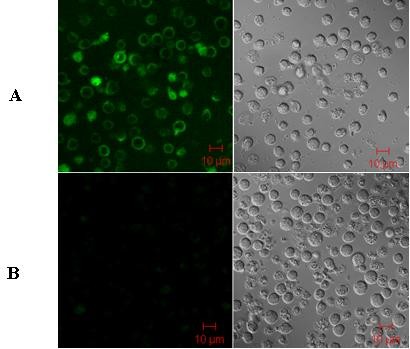
**Antigen-specific TCR gene-transduced CD3+T cells from PB of healthy individuals stained with TCR Vβ13-specific antibody and imaged by LSM (×630). A) CD3+T cells transduced with Vβ13-IRES-TCR Vα6 recombinant plasmid and stained by FITC-TCR Vβ13-specific antibody**. B) CD3+T cells transferred with empty vector as a negative control for exogenous TCR Vβ13 expression.

**Figure 4:**
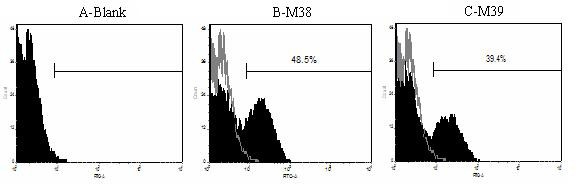
**The use of two TCR vector cassettes results in differential expression in human CD3+T cells**. TCR gene-transduced CD3+T cells were stained with a TCR Vβ13-specific antibody and analyzed by flow cytometry. The numbers indicate the percentage of TCR Vβ13-positive cells. A) Human CD3+T cells transferred with empty vector as a negative control for exogenous TCR Vβ13 expression. B) Human CD3+T cells transferred with TCR Vβ13-IRES-TCR Vα6 recombinant plasmid were stained with a TCR Vβ13-specific antibody 48 h after transduction. C) Human CD3+T cells transferred with TCR Vα6-IRES-TCR Vβ13 recombinant plasmid were stained with a TCR Vβ13-specific antibody 48 h after transduction. The TCR Vβ13 gene specific for DLBCL exhibited higher expression in TCR gene-transferred CD3^+^T cells when the βIα vector construct was used.

Human CD3+T cells were transduced with the recombinant plasmids harboring the two transgenic cassettes. Immunophenotyping analysis 48 h after transduction was performed by LSM using TCR Vβ13 mAbs (Figure 3). The gene transfer efficiency was assessed by CD3+T cell staining with antibodies directed against TCR Vβ13 by FCM 2 days after transduction (Figure 4). TCR α-chain expression was not detected due to the lack of a Vα6-specific antibody. TCR CD3+T cells that were not transduced served as a negative control. The percentage of TCR gene-transduced cells revealed differences in gene expression levels between the αIβ transgene cassette and the βIα transgene cassette. The surface expression of exogenous TCRβ from cassette βIα (48.5%; Figure 4B) was superior to that of cassette αIβ (39.4%; Figure 4C), compared to ~1% in the control cells (Figure 4A). This is most likely due to the poor expression of the TCR β-chain gene in the 3' position of the IRES linker relative to the insertion in the 5' position, although there was no statistical significance between the gene expression in the 3' and 5' positions of the IRES (*P *= 0.21). Nevertheless, this still indicates that the 3' position of an IRES element is an unprivileged position leading to suboptimal TCR chain expression levels.

### Functional tests of TCR transductants

**Figure 5:**
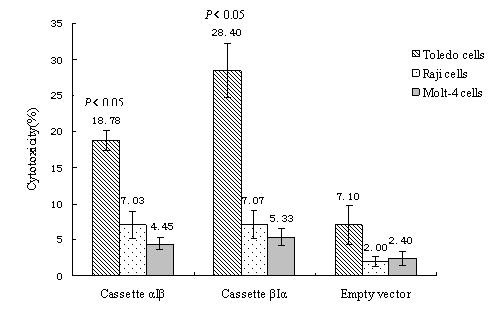
**Specific cytotoxicity of TCR gene-transduced CD3+T cells directed against Toledo cells as determined by LDH release assay**. Three days after transduction, TCR gene-transduced CD3+T cells by two different TCR transgene cassettes were cocultured with Toledo, Raji, or Molt-4 cells at a 10:1 ratio for 10 h. Then, the LDH level in the supernatant was determined. The spontaneous release of LDH from both target and effector cells was subtracted from the measured values and the final results are expressed as the percentage of specific cytotoxicity. *Mann-Whitney test of two independent samples was used to determine differences between the various groups. Statistical significance was defined as *P *< 0.05.

Finally, we analyzed whether the surface expression levels of exogenous TCR α or β achieved by the different vector cassettes influence TCR function. Cassette βIα achieved a higher transduction efficiency than did cassette αIβ. However, the positive cell populations of about 48.5% (TCR Vβ13 single-positive cells) with cassette βIα cannot exclude the presence of mixed TCR heterodimers having formed as the transgenic TCR chains mispaired with endogenous chains. To further characterize the function of TCR, we evaluated the impact of these two different transgene cassettes on the specific cytotoxicity of TCR gene-transduced CD3+T cells. The cytotoxicity of TCR gene-transduced CD3+T cells was determined using a nonradioactive LDH release assay. The LDH level was analyzed following the cocultivation of effector cells (TCR gene-transduced CD3+T cells or CD3+T cells transduced with empty plasmid) with target cells (Toledo, Molt-4, and Raji cells). The specific cytotoxicity of the TCR gene-transduced CD3+T cells against Toledo cells by cassette βIα was higher than that observed with cassette αIβ (*P *= 0.014), suggesting that the TCR transgene vector βIα yielded a better function of human antigen-specific TCR-redirected T cells than did vector αIβ. Cytotoxicity of TCR gene-transduced CD3+T cells against Toledo cells by two types of TCR transgene cassettes (αIβ, *P *= 0.008; βIα, *P *= 0.000) was significantly higher than that of CD3+T cells transduced with empty vector. From these cocultivations of effector cells with target cells, we found that the cocultivation of TCR gene-transduced CD3+T cells with the Toledo cell line achieved the highest LDH level (Figure 5), indicating that TCR genes-transduced CD3+T cells were specifically directed against the DLBCL cell line.

## Discussion

The successful transfer of genes encoding TCRαβ chains, which recognize a variety of virus-specific and tumor-associated antigens, into primary T cells was demonstrated previously [11,12,24,25]. For clinical applications of TCR-redirected T cells, the efficient functional expression of the transgenic TCR is a prerequisite. The selection of an optimal transgenic cassette offers a simple option to enhance functional TCR expression, as well as a means to explore more complex modifications of TCR chain genes to obtain preferential pairing (murinization, additional cysteine bonds) [26,27] or enhanced expression through codon modification [28].

In general, TCR α- and β-chain genes can be commonly linked by an IRES. Because a gene inserted downstream of the IRES will be expressed at significantly lower levels than one introduced to the upstream position,^23 ^and the two chains may play nonequivalent roles in antigen selection, we integrated TCRα into either the IRES 5' position (TCRVα6-IRES-Vβ13) or the 3' position (TCRVβ13-IRES-Vα6), and generated two types of recombinant plasmids. Subsequently, we compared the influence of the transgenic cassettes on the expression and function of the TCR specific for DLBCL-associated antigen and found that the application of cassette βIα resulted in higher expression and functionality of a human TCR when compared to that observed with the use of cassette αIβ. Yet, if the P2A element (2A element of porcine teschovirus) [29,30], instead of the IRES element, were employed to link a single TCRα- and β-chain-encoding mRNA, then plasmid TCRβ-P2A-TCRα may achieve higher TCR chain expression and T cell function compared to the TCRβ-IRES-TCRα plasmid [31]. A potential problem with using the P2A linker is that parts of the virus-derived sequence might be presented by MHC I molecules, making the transferred T cells a target for elimination by the host's immune system [31]. These findings may have serious consequences for the design of TCR expression cassettes, which are used to change the antigen specificity of T cells employed for adoptive T cell therapy.

Most of the known T cell-recognized epitopes are those presented by MHC class I molecules to CD8+T cells, and relatively few MHC class II tumor epitopes have been identified. Thus, to date, most adoptive immunotherapy approaches have focused on CD8^+ ^CTL. However, the ability to transfer TCR genes between T cells now means that both CD4^+ ^and CD8^+ ^lymphocytes can be generated against the same specific targets, offering concerted therapeutic strategies that can fully utilize adoptive transfer [32-34]. Thus, in the current study, we screened CD3+T lymphocytes (including CD3+CD4+and CD3+CD8+T cells) as the recipient cells of TCR gene transduction. In human subjects, normal autologous T lymphocytes, transduced *ex vivo *with anti-tumor associated antigen (TAA) and the TCR genes, which were re-infused into cancer patients, persist and express the transgene for a prolonged time *in vivo *and mediate the durable regression of large established tumors [19]. Ideally, TCR genes specific for DLBCL-associated antigen should be transduced into autologous T lymphocytes, which aim directly at autologous lymphoma cells. It is a pity that we were unable to obtain patients' autologous lymphoma cells as target cells. With respect to the most commonly distributed human MHC I molecule HLA-A2, we selected an HLA-A2-positive DLBCL cell line (Toledo cell line) as the target cells. Antigen-specific TCR gene-modified T cells acquired the ability for DLBCL-specific cytotoxic capacity according to *in vivo *cytotoxicity assays. The effect of the TCR Vβ13-IRES-TCR Vα6 recombinant plasmid-transferred T cells was superior to that of the TCR Vα6-IRES-TCR Vβ13-transferred T cells; specifically, the cytotoxicity of TCR-transferred T cells with cassette βIα directed to the DLBCL cell line arrived at 30% 72 h after transduction, however, the TCR-modified T cells showed lower cytotoxicity for Molt-4 and Raji cells, which do not express DLBCL-associated antigen; the specific anti-DLBCL activity might be confirmed in this study. As tumor cells possess more than one TAA, it is possible that there are multiple T cell clones specific for tumor cells. Further genetic modification of PBLs with a few specific TCRs may be beneficial.

This study suggests the therapeutic potential of genetically engineered cells for the biologic therapy of cancer. However, in the present study, as first step to investigate the expression and the effect of TCR Vα6/Vβ13 recombinant vectors in vitro, we used the transient expression technique, in which the genes could expression only few day after transfection, and we could conform that specific anti-DLBCL cytotoxic T lymphocyte (CTL) could be induced by transduction of specific TCR gene to modify healthy T cells. For the clinical application in future, stable expression of transduced genes in T cells is necessary and should be optimized the transfection technique, maybe using different viral vectors.

In summary, to our knowledge, this is the first demonstration of DLBCL-associated antigen-specific TCR gene-modified T cells having acquired specific cytotoxicity. However, the present study should be followed by a large cohort of cytotoxicity assays for different DLBCL cell lines to confirm its common anti-DLBCL cytotoxicity, which was thought to target the TAA of DLBCL. This study provides substantial new data for a better understanding of the strategy of adoptive immunotherapy in DLBCL.

## Competing interests

The authors declare that have no competing interests.

## Authors' contributions

QSY performed TCR gene cloning and transfer and data management, LJY performed T-cells culture, SHC performed the RT-PCR and genescan analysis, YBZ performed the western blot, XLW and XFZ performed the real-time PCR, YQL were responsible for the study design and data management. All authors read and approved the final manuscript.
